# Reliability of velocity pulsatility in small vessels on 3Tesla MRI in the basal ganglia: a test–retest study

**DOI:** 10.1007/s10334-022-01042-2

**Published:** 2022-09-27

**Authors:** Rick J. van Tuijl, Stanley D. T. Pham, Ynte M. Ruigrok, Geert Jan Biessels, Birgitta K. Velthuis, Jaco J. M. Zwanenburg

**Affiliations:** 1 Center for Image Sciences, Radiology Department, University Medical Center Utrecht, Utrecht, The Netherlands; 2 Department of Neurology and Neurosurgery, UMC Utrecht Brain Center, Utrecht, Netherlands; 3 Department of Radiology University Medical Center Utrecht, Utrecht University, Utrecht, The Netherlands

**Keywords:** Velocity pulsatility, Basal ganglia, Test–retest study, 3Tesla MRI, 2-dimensional phase-contrast

## Abstract

**Objective:**

Recent work showed the feasibility of measuring velocity pulsatility in the perforating arteries at the level of the BG using 3T MRI. However, test–retest measurements have not been performed, yet. This study assessed the test–retest reliability of 3T MRI blood flow velocity measurements in perforating arteries in the BG.

**Materials and methods:**

Two-dimensional phase-contrast cardiac gated (2D-PC) images were acquired for 35 healthy controls and repeated with and without repositioning. 2D-PC images were processed and analyzed, to assess the number of detected perforating arteries (*N*_detected_), mean blood flow velocity (*V*_mean_), and velocity pulsatility index (vPI). Paired *t*-tests and Bland–Altman plots were used to compare variance in outcome parameters with and without repositioning, and limits of agreement (LoA) were calculated.

**Results:**

The LoA was smallest for *V*_mean_ (35%) and highest for vPI (79%). Test–retest reliability was similar with and without repositioning of the subject.

**Discussion:**

We found similar LoA with and without repositioning indicating that the measurement uncertainty is dominated by scanner and physiological noise, rather than by planning. This enables to study hemodynamic parameters in perforating arteries at clinically available scanners, provided sufficiently large sample sizes are used to mitigate the contribution of scanner- and physiological noise.

## Introduction

Recently developed MRI methods directly assessed vessel pulsatility and blood flow velocity in the perforating arteries as hemodynamic parameters that are potentially relevant to cerebrovascular diseases, such as small vessel disease (SVD) [1–3]. SVD is common among elderly and contributes to cognitive decline and dementia. Pulsatility measurements could be of interest for understanding its pathophysiology. SVD pathologies lead to white matter hyperintensities, lacunar infarcts, enlarged perivascular spaces, microbleeds, and subcortical infarcts, which can be visualized by using MRI [4–6]. However, these MRI-visible tissue lesions represent the irreversible end-stage of SVD. Moving away from these markers of irreversible tissue damage to markers that more specifically reflect small vessel function is urgently needed. Markers of small vessel function would, first, enable mechanistic research into the mechanisms that relate SVD to clinically relevant outcomes such as stroke and dementia, which could stimulate the development of new treatments. Second, these markers have the potential to evolve into imaging biomarkers that can be used in clinical trials for the assessment of the efficacy of newly developed treatments, and/or in a clinical setting for better characterization of the disease severity.

Previous studies have shown that pulsatility is higher in the BG in patients with lacunar infarction than in healthy controls [7] and that pulsatility increased with age in the lenticulostriatal arteries supplying the BG on 7T MRI [7, 8]. These markers have the potential to evaluate aspects of vascular dysfunction before the development of irreversible macroscopic brain damage associated with SVD. These parameters can be measured on 7T MRI [9]; however, 7T MRI is not routinely used in standard clinical care and has very limited availability worldwide. The sensitivity of 3T MRI is lower compared to 7T MRI, but much more widely available. The ability to assess hemodynamic parameters of the perforating arteries on standard clinical 3T MRI would allow for widespread use in clinical studies regarding SVD, which would accelerate the evaluation of the value of these measurements in research and clinical care. Recent work showed the feasibility of measuring velocity pulsatility in the perforating arteries at the level of the basal ganglia using 3T MRI [9]. However, test–retest measurements have not been performed on 3T MRI, but are necessary for better insight into the robustness of the outcome measurement and statistical power calculations in future studies.

This study aimed to assess the reliability of 3T MRI velocity measurements in perforating arteries by performing test–retest scans. Scans were repeated with and without repositioning in order to allow for assessing the relative contribution of the planning uncertainty to the measurement errors.

## Methods

### Data availability

Anonymized data will be shared upon reasonable request to the corresponding author.

### Study participants

We aimed to include 35 subjects in this study to estimate the standard deviation of the test–retest variability within 20% of its true values with 90% confidence [10]. Subjects were only included if they did not have cerebrovascular disease according to their self-reported medical history. One subject had to be excluded because a large intracranial aneurysm was diagnosed as an incidental finding on the 3D T_1_-weighted anatomical scan. This subject was replaced by a new participant, maintaining the number of 35 included subjects. The local ethics review committee approved the study and written informed consent was obtained from all subjects.

### MRI acquisition

**Fig. 1:**
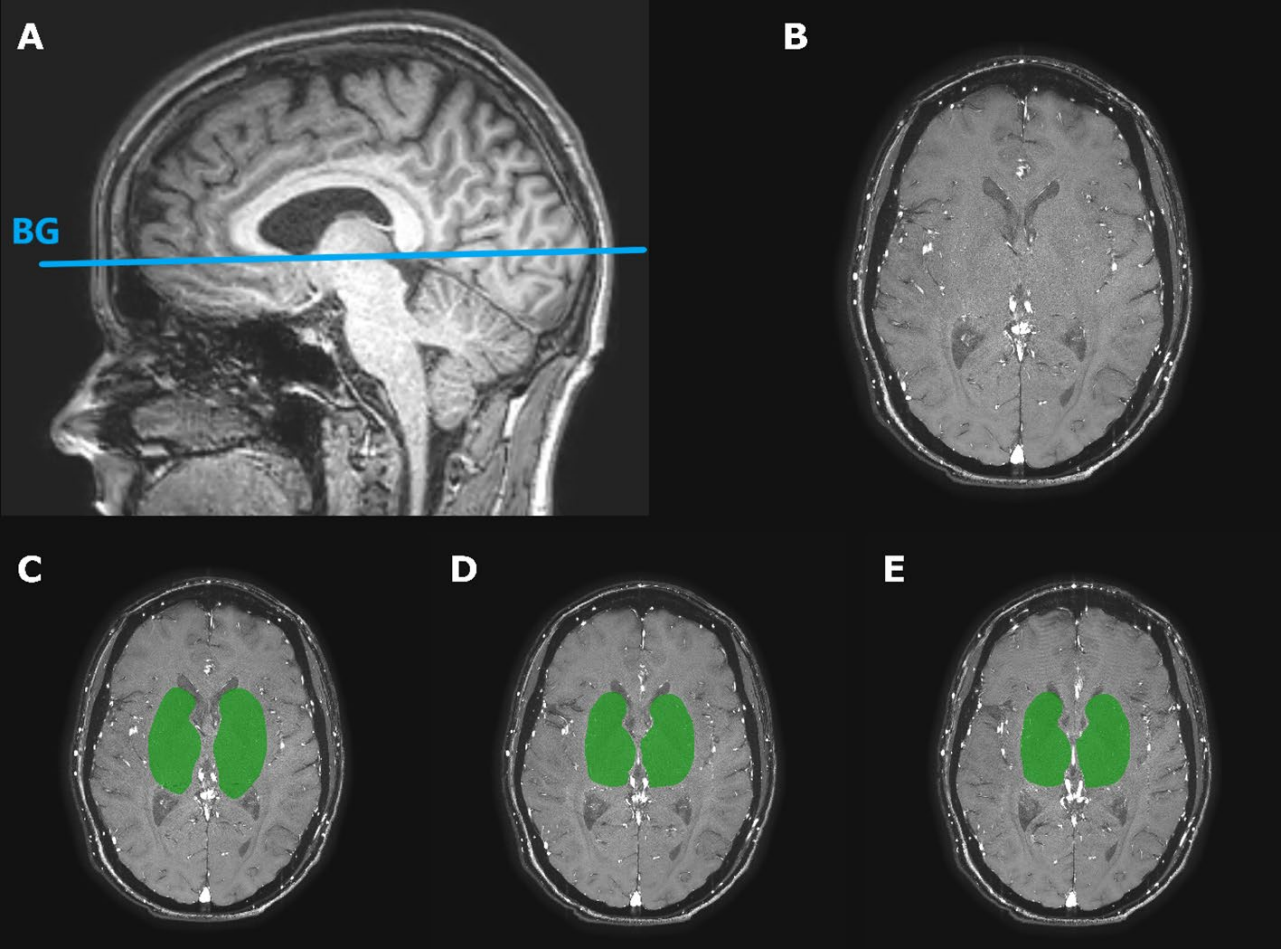
Illustration of the slice planning and the used region of interest (ROI). **A** The 2-dimensional phase contrast angiogram (2D-PC) magnetic resonance imaging slice through the basal ganglia (BG) was planned parallel to the genu and splenium of the corpus callosum, on a 3D T1-weighted image. **B** Magnitude image of a representative 2D-PC BG slice of a subject, after planning the image as shown in A. **C** ROI as shown in green for the first 2D-PC scan. The ROI was manually drawn between the insula and the ventricles, avoiding sulci and cortical gray matter. **D** The second 2D-PC including the drawn mask, which represents the ROI. The second 2D-PC was obtained after repositioning of the subject after a 5 min break. **E** The third 2D-PC including its ROI. This third 2D-PC BG was scanned immediately after the second 2D-PC, without repositioning the subject or replanning the slice, to allow for additional analysis of the test–retest reliability without repositioning

Blood flow velocity of the small perforating arteries at the level of the BG was measured using a previously published velocity encoded 2-dimensional phase contrast (2D-PC) acquisition performed at 3 T MRI with a 32-channel head coil (3 T Ingenia Elition, Philips Healthcare, Best, The Netherlands) [9]. The 2D-PC acquisition was planned on a 3D T1-weighted anatomic acquisition at the level of the BG (Fig. 1). The 2D-PC sequence was retrospectively gated using a peripheral pulse-oximeter for triggering. The following scan parameters were used: 250 × 250 mm^2^ field of view; acquired spatial resolution 0.3 × 0.3 × 2.0 mm^3^, reconstructed spatial resolution (through zero-filling in k-space) 0.2 × 0.2 × 2.0 mm^3^; TR/TE = 28/14.5 ms; flip angle = 50°; readout bandwidth = 44 Hz/pixel; velocity encoding = 20 cm/s; acquired temporal resolution = 168 ms; 8–15 reconstructed heart phases, depending on the heart rate; sensitivity encoding factor = 1.5; scan duration was about 3 min for a heart rate of 60 beats/min. Scans were visually evaluated for subject motion and repeated if necessary. Substantial subject motion was recognized by motion artifacts on the 2D PC images, such as overt blurring and/or intensity inhomogeneity that varies over the cardiac cycle (spatio-temporal waves). Four scans in four different subjects were repeated, two times the first scan, once the repeated scan with repositioning, and once the repeated scan without repositioning.

### Test–retest reliability

Subjects were scanned in the head-first, supine position. After the initial positioning of the subject, the first 2D-PC MRI scan was acquired. All subjects were briefly taken out of the scanner and repositioned after a short break of 5 min. After repositioning, a second 2D-PC scan was acquired for assessing test–retest reliability with repositioning. A third 2D-PC scan was acquired without repositioning and replanning (Fig. 1) to assess test–retest reliability without repositioning. Repeating the 2D-PC acquisition both with and without repositioning allows to assess the relative contributions of variability due to thermal- and physiological noise, and the variability due to variability in planning.

### Image processing

The 2D-PC images were processed and analyzed to assess the number of detected perforating arteries (*N*_detected_), their average mean blood flow velocity (*V*_mean_) determined by averaging over *N*_detected_ and their average velocity pulsatility index (vPI) obtained from the mean normalized velocity curve. Analysis of the 2D-PC images was performed as described previously [2, 3]; however, the algorithm has been re-implemented in a Python-based tool for better user friendliness and easier maintenance. The Python-based analysis tool was used to process the images and detect the perforating arteries. We made the code used to segment and analyze the small perforating arteries publically available (https://github.com/VBIG-UMCU/SELMA). In brief, this tool comprised the following steps: First, a region of interest (ROI) of the BG on the 2D-PC image was manually drawn in the user interface of the tool (Fig. 1). In this ROI, the noise level was estimated on a pixel-by-pixel basis, using the standard deviation of the (complex) signal over the cardiac cycle. For this 3 T data, pixels with an estimated SNR in the mean magnitude image (i.e. average over the cardiac cycle) of 2 or less were subsequently removed from the ROI. Next, the SNR of the mean velocity was estimated based on the estimated noise level in the magnitude images. Pixels with a mean velocity significantly above the noise level were clustered together if adjacent to each other and all clusters were marked as potential perforating arteries. As perforating arteries generally have diameters smaller than the voxel size, the voxel with the highest mean velocity in such a cluster was selected as representative for the perforating artery in case of clusters of adjacent voxels with significant mean velocity. Non-perpendicular arteries were removed by assessing the circularity of the cluster shape. Arteries with a ratio of the largest axis length and the smallest axis length larger than a set threshold of 2 were considered to be elliptical with a non-perpendicular orientation to the scanning plane and thus were removed. Finally, arteries in separate clusters within 1.2 mm distance from each other were assessed and the artery with the highest velocity was kept. Multiple potential arteries within this close proximity to each other are mostly false positive voxels, e.g. due to ghosting of the detected vessel. Our previous publications describe the implemented algorithm in more detail [2, 9].

**Fig. 2:**
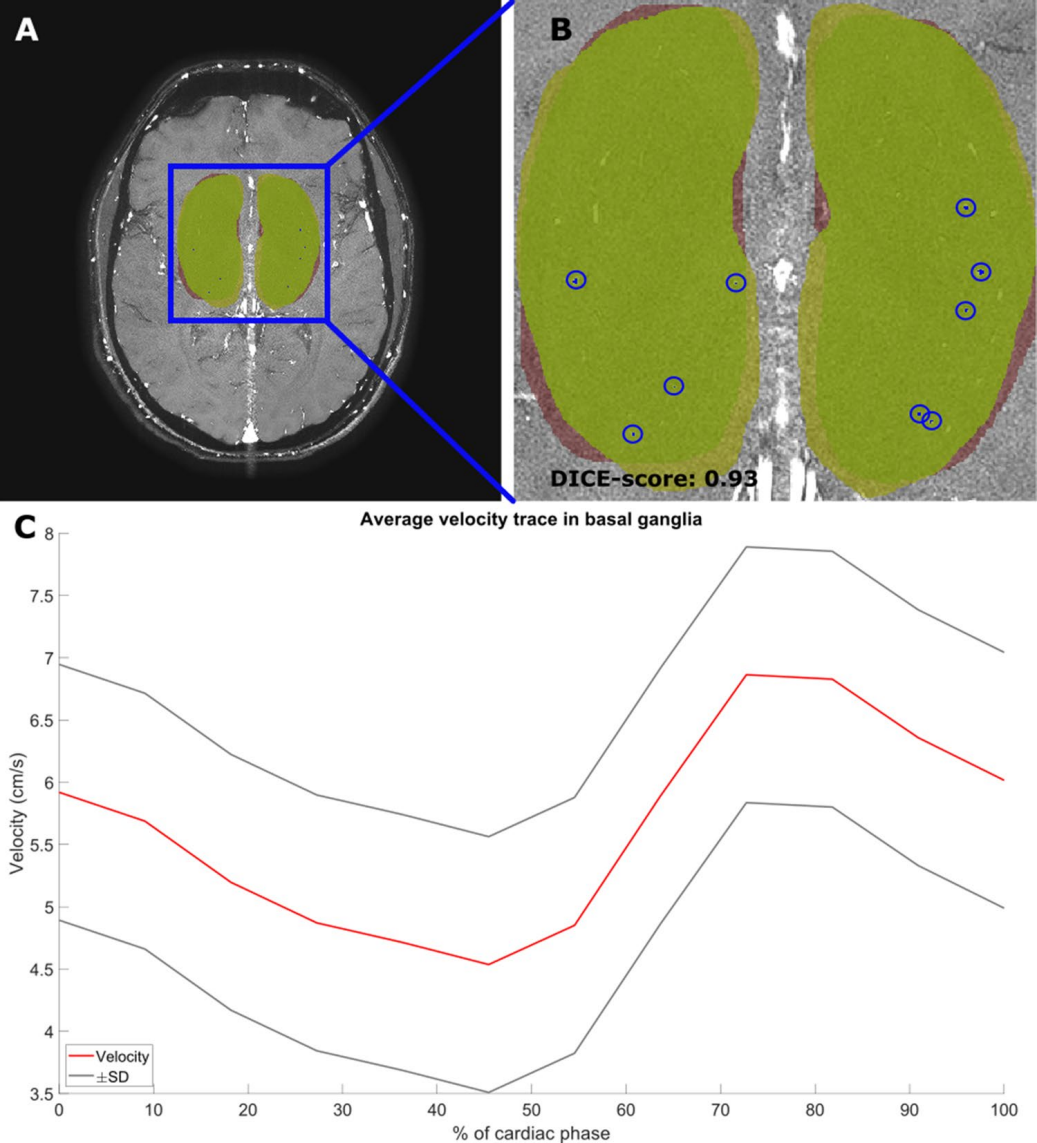
Illustration of the DICE coefficient calculation and region of interest (ROI) drawings by the two observers. **A** The 2-dimensional phase contrast angiogram (2D-PC) magnetic resonance imaging slice through the basal ganglia (BG), with the two drawn regions of interest (ROIs) by the two observers (red and green). **B** Zoomed in on the ROIs whereby the blue circles mark the detected perforating arteries by the Python tool. This image shows that all found perforating arteries are localized in both manually drawn ROIs. **C** The average mean velocity waveform of the perforating arteries (red line), with the standard error of the mean visualized (black lines). Since the same number of perforating arteries are found by both observers, the velocity waveform is also the same

**Fig. 3:**
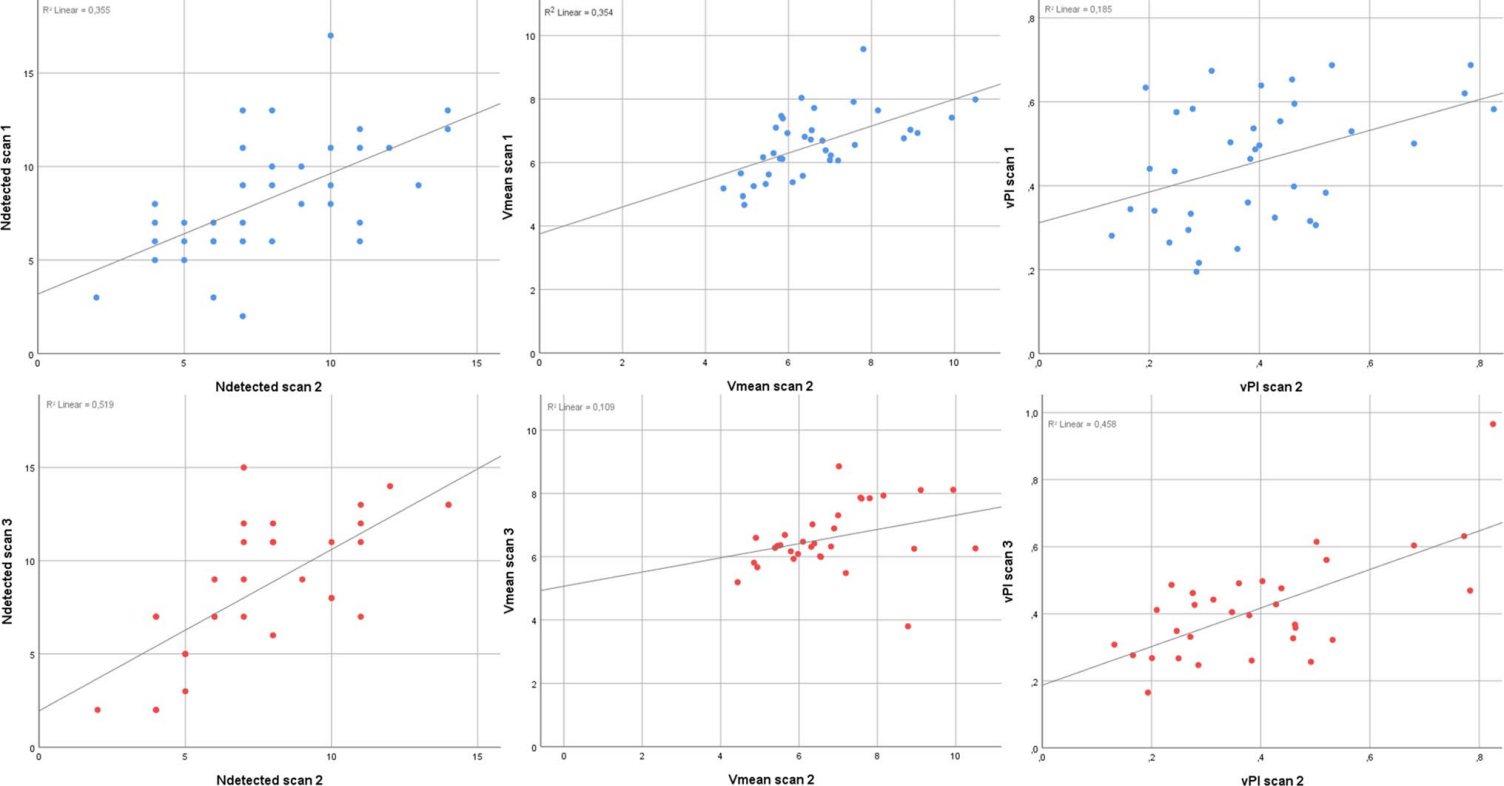
Scatterplots of *N*_detected_, *V*_mean_ and vPI. Top row shows the scatterplots between the first (*Y*-axis) and second scan (*X*-axis) in blue for, from left to right, *N*_detected_ (ICC = 0.82, *p* < 0.001), *V*_mean_ (ICC = 0.78, *p* < 0.001) and vPI (ICC = 0.73, *p* = 0.008), respectively. The bottom row represents the outcome measurements between the second (*X*-axis) and third scan (*Y*-axis) in red for *N*_detected_ (ICC = 0.82, *p* < 0.001), *V*_mean_ (ICC = 0.81, *p* < 0.001) and vPI (ICC = 0.71, *p* = 0.001), respectively

The velocity waveform was extracted over the entire cardiac cycle for every vessel (Fig. 2). The mean overall velocity of the scan (*V*_mean_) was then defined as the mean velocity of the average velocity waveform over all vessels (Fig. 3). For the calculation of the vPI, the velocity curve of every vessel was first normalized before averaging over all vessels. From the resulting mean normalized velocity curve over all *N*_detected_, the vPI was calculated using the following established definition as given in Eq. (1) [11]:

1 $${\text{vPI}} = \frac{{V_{ \max } - V_{ \min } }}{{V_{{\text{ mean}}} }}$$

Here, *V*_mean_ is the mean of the mean normalized velocity curve, which is 1 as a result of the normalization procedure. *V*_max_ and *V*_min_ were the respective maximum and minimum of the average velocity waveform over the cardiac cycle of the mean normalized velocity curves.

### Inter-rater reliability

The 2D-PC acquisition was planned on a 3D T1-weighted anatomic acquisition at the level of the BG where we manually draw an ROI to delineate the BG. The drawn ROI at the level of the BG were separately drawn for the left and right hemispheres. For each hemisphere, the BG ROI was bordered on the medial side by the interhemispheric fissure and on the lateral side by the cortical gray matter of the insula (excluding cortical gray matter). The anterior border was formed by the anterior horn of the lateral ventricles and the edge of the caudate nucleus. The posterior border was formed by the posterior border of the thalamus. The BG ROIs were adjusted to exclude tissue areas where an intrasulcal vessel ghosted over the tissue. The ROIs were drawn by two independent researchers (RT and SP, after RT trained SP on drawing ROIs at the BG). Inter-rater reliability was determined based on the first scans all 35 subjects. Similarity of the ROIs was determined by calculating the Dice coefficient (DC) [12]. DC is calculated as DC(A,B) = 2(A ∩ B)/(A + B) where ∩ is the intersection between ROI A and ROI B. The reliability of the drawn ROIs and the reliability of the outcome measurements *N*_detected_, *V*_mean_, and vPI was determined.

### Statistical analysis

All statistical analyses were performed in IBM Statistics Package of Social Sciences (SPSS) Version 25 (Chicago, IL, USA). First, normality of data was tested using the Shapiro–Wilk test of normality. Next, we determined whether statistically significant differences existed between test–retest reliability with and without repositioning in *N*_detected_, *V*_mean_, and vPI using a paired Student’s *t*-test. A *p*-value < 0.05 was considered statistically significant. This test was performed to study potential physiological effects. Scatterplots were created to visually relate the measurement results from the first and second scan (repeats with repositioning), and the second and third scan (repeats without repositioning). Intraclass correlation coefficients (ICC) were computed as a reliability measurement.

Bland–Altman [13, 14] plots were used to compare variance in outcome parameters with and without repositioning and limits of agreement (LoA) were calculated from the mean differences and the standard deviation of the differences [15]. The coefficient of repeatability (CoR) represents the value below which the absolute difference between two repeated measurements may be expected to lie with a probability of 95%. The CoR was computed as 1.96 * the standard deviation of the differences [13] and presented as percentage relative to the mean measured values. A large variance in outcome parameters with repositioning compared to the mean values suggests that the measurement noise dominates over the variance in outcome parameters with repositioning, resulting in limited agreement between the scans. Conversely, a small variance in outcome parameters without repositioning compared to the mean values implies a high level of agreement between the two scans.

## Results

### Inter-rater reliability

The average DC between the drawn ROIs for all 35 subjects by the trained operators RT and SP was 0.90 (range between 0.85 and 0.95). The calculated *N*_detected_, *V*_mean_, and vPI for the ROIs of both observers are given in Table 1.

**Table 1:** Interrater reliability (*n* = 35 scans)

	Operator 1	Operator 2	Percentage deviation (%)
Number of perforating arteries	8 (7–9)	8 (7–9)	9 (4–14)
Mean velocity (cm/s)	6.6 (6.1–7.1)	6.6 (6.1–7.1)	3 (2–5)
vPI	0.40 (0.34–0.46)	0.40 (0.34–0.45)	9 (3–14)

Values given in mean with 95% confidence intervals

Percentage deviation: [abs(Operator 1 Operator 2)/Operator 2] * 100%

### Test–retest reliability

Suitable datasets for test–retest reliability with repositioning (first and second scans) were available for all 35 subjects. Test–retest reliability without repositioning (second vs third scan) were available in 30 subjects, as this scan was added later, once it was clear that there was sufficient scan time for this additional test. The group consisted of 18 men (51%) and 17 women and had a mean age of 28 ± 10 years. Perforating arteries were successfully detected in all subjects. The mean and standard deviations of *N*_detected_, *V*_mean_, vPI and heart rate for the three different measurements are given in Table 2 and all three outcome measurements were normally distributed for all three scan times. Scatterplots of *N*_detected_, *V*_mean,_ and vPI. Top row shows the scatterplots between the first (*Y*-axis) and second scan (*X*-axis) in blue for, from left to right, *N*_detected_ (ICC = 0.82, *p* < 0.001), *V*_mean_ (ICC = 0.78, *p* < 0.001), and vPI (ICC = 0.73, *p* = 0.008), respectively. The bottom row represents the outcome measurements between the second (*X*-axis) and third scan (*Y*-axis) in red for *N*_detected_ (ICC = 0.82, *p* < 0.001), *V*_mean_ (ICC = 0.81, *p* < 0.001), and vPI (ICC = 0.71, *p* = 0.001), respectively.

**Table 2:** Outcome parameters of the 2D phase-contrast (2D-PC) scans

	First 2D-PC image	Second 2D-PC image	*P*-value scan 1 vs. scan 2	Third 2D-PC image	*P*-value Scan 2 vs. scan 3
Number of scans	35	35	–	30	–
Heart rate in beats per minute	68 ± 10	66 ± 10	0.41	66 ± 10	0.84
Number of perforating arteries	8 ± 3	8 ± 3	0.68	9 ± 4	0.43
Mean velocity (cm/s)	6.5 ± 1.0	6.6 ± 1.4	0.84	6.5 ± 1.0	0.85
Velocity Pulsatility Index	0.45 ± 0.14	0.40 ± 0.17	0.18	0.41 ± 0.16	0.74

Values are given as mean ± standard deviation

Second 2D-PC image after 5 min break repositioning and replanning. Third 2D-PC image was directly repeated after 2nd scan without repositioning or replanning

### Test–retest reliability with repositioning

**Fig. 4:**
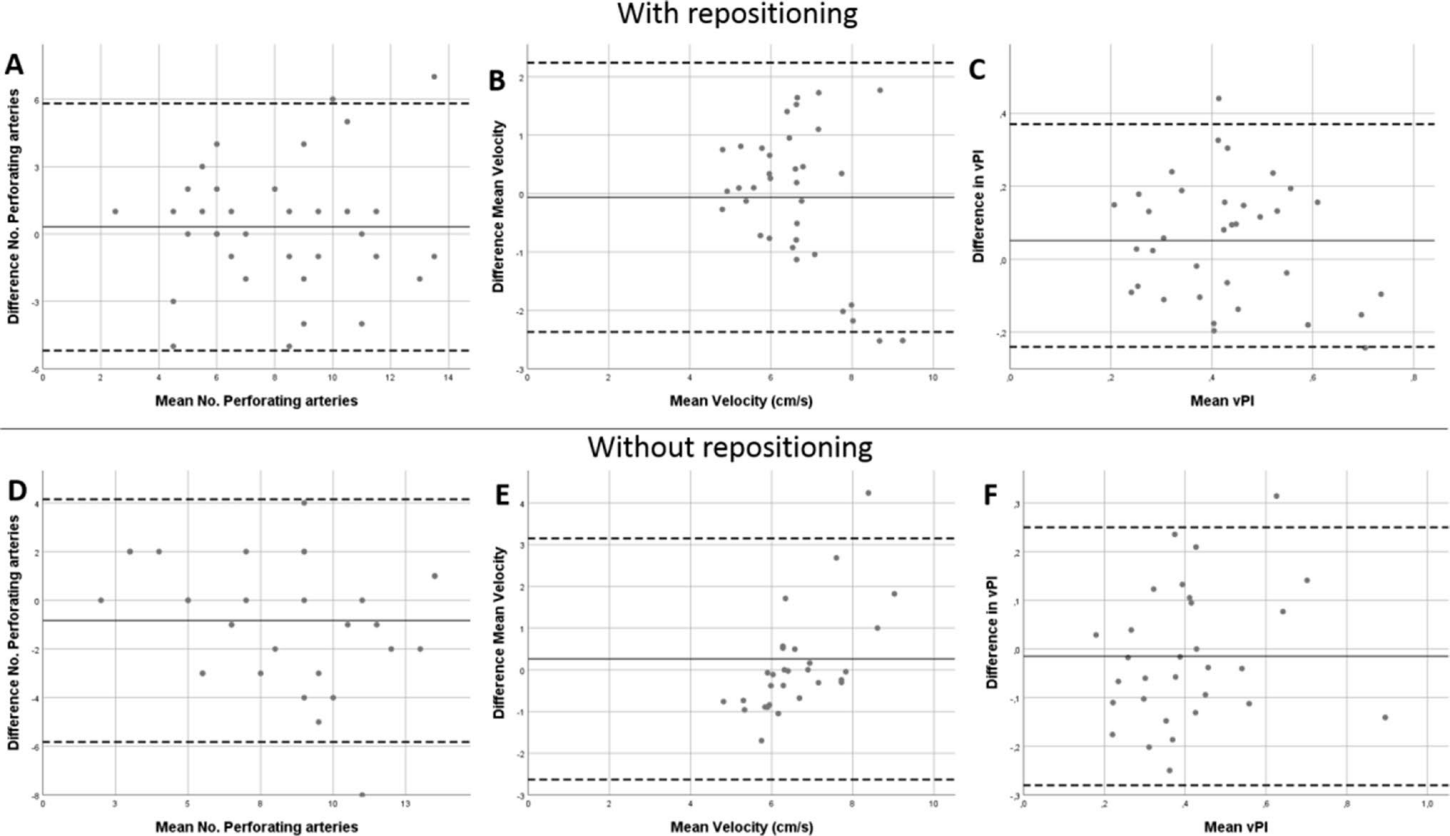
Bland–Altman plots for test-retest reliability with and without repositioning comparison for all outcome measurements. The bold line shows the mean in every figure and the dashed lines show the 95% Limits of Agreement. With repositioning for (**A**) number of perforating arteries included, (**B**) mean velocity and (**C**) velocity pulsatility index (vPI). Without repositioning for (**D**) number of perforating arteries included, (**E**) mean velocity and (**F**) vPI

Results were very similar comparing the first and second 2D-PC scan. *N*_detected_ was the same in both the first scan 8 ± 3, and after repositioning 8 ± 3.*V*_mean_ was also similar between the first and second 2D-PC scan (6.5 ± 1.0 cm/s and 6.6 ± 1.4 cm/s, respectively). vPI was higher in the first 2D-PC scan 0.45 ± 0.14 compared to the second 2D-PC scan 0.40 ± 0.17, although this was not significant (*P* = 0.18). The CoR, expressed as percentage of the mean values, were 68%, 35%, and 79% for *N*_detected,_ *V*_mean,_ and vPI, respectively. The mean measurement and the mean, standard deviation (SD), and LoA of the difference obtained from the Bland–Altman analysis between the two scans can be found in Table 3 and Fig. 4.

**Table 3:** Descriptive statistics from the Bland–Altman analysis for test-retest reliability with and without repositioning

Difference in	*N*	Mean measurement	Mean difference	Std. Deviation of difference	LoA
With repositioning					
* N*_detected_	35	8	0.31	2.81	5.19–5.81
* V*_mean_	35	6.58	0.06	1.18	2.37–2.24
vPI	35	0.43	0.05	0.16	0.27–0.37
Without repositioning					
* N*_detected_	30	8	0.83	2.54	5.82–4.15
* V*_mean_	30	6.61	0.26	1.48	2.63–3.15
vPI	30	0.41	0.01	0.14	0.28–0.26

*LoA* Limits of Agreement, *N* number of scans

### Test–retest reliability without repositioning

Results were very similar comparing the two acquired 2D-PC images (second and third scan) without repositioning: *N*_detected_ = 8 ± 3, *V*_mean_ = 6.6 ± 1.4 cm/s and vPI = 0.40 ± 0.17 vs *N*_detected_ = 9 ± 4, *V*_mean_ = 6.5 ± 0.9 cm/s and vPI = 0.41 ± 0.16 (Fig. 4). The CoR, expressed as percentage of the mean values, were 60%, 42%, and 61% for *N*_detected,_ *V*_mean_ and vPI, respectively.

## Discussion

In this study, we assessed the test–retest reliability of assessing perforating arteries of the basal ganglia with 2D-PC velocity measurements at 3 T MRI, with and without repositioning. The CoRs of *N*_detected_, *V*_mean_, and vPI with repositioning are in the same range of those obtained without repositioning. The standard deviations of the differences in *N*_detected_, *V*_mean_ and vPI between repeated scans, were similar compared to the respective inter-subject standard deviations, which indicates that uncertainty in measuring the amount of detected vessels was dominated by scanner and physiological noise, rather than by planning and inter-subject variation. The CoR was lowest for *V*_mean_ (35%) and highest for vPI (79%). The interrater reliability assessment showed good consistency between two raters with mean variability below 10% for all parameters. Since we have relative low *N*_detected_, a small difference of drawn ROI between two observers could introduce relative big differences in *V*_mean_ and vPI. The obtained results can be used for power calculations for future 3 T MRI studies.

The values we found in the first scan are in line with another study with similar age but relatively more man than women (10 vs. 5) compared to our group [9]: *N*_detected_ 5 ± 3 in [9] (versus 8 ± 3 in our study), *V*_mean_ 6.0 ± 1.3 cm/s (versus 6.5 ± 1.0 cm/s) and vPI 0.49 ± 0.19 (versus 0.45 ± 0.14). Our CoR results are higher compared to literature for pulsatility for the lenticulostriatal arteries and middle cerebral artery for young subjects (37% and 42%, respectively; mean age 25 ± 3 years) and for old subjects (35% and 35%, respectively; mean age 75 ± 5 years) at 7 T MRI [8]. The CoR for the pulsatility and *V*_mean_ at the BG obtained at 7 T MRI in another study were 38%, and 41%, respectively [1]. The higher CoR of our study compared to the 7 T results from the literature show that CoR for outcome parameters, *N*_detected_, *V*_mean,,_and vPI, are higher at 3 T MRI than at 7 T MRI. This is not surprising given the reduced sensitivity of 3 T [9]. The relative uncertainty in the previously reported 3 T measurements of the vPI of the perforating arteries (i.e. vPI = 0.45 ± 0.19 [9]) was 83%, if we assume that the standard deviations in these measurements were dominated by measurement noise rather than by physiological inter-subject variation. This value is in the same range as the values we report in the current study. However, as discussed in recent work directly comparing 3 T and 7 T measurements [9], the vPI measurements at 7 T tend to be lower as the higher sensitivity at 7 T leads to the inclusion of additional smaller vessels that have lower velocities, and lower pulsatility indices. Partial volume effects have a non-linear effect on the velocity waveform, and lead generally to overestimation of the observed vPI [1]. This overestimation is expected to be higher at 3 T MRI than at 7 T MRI due to better background signal suppression at 7 T resulting from the longer T1 relaxation time constant of tissue at higher field strength [9].

The European Society of Radiology has written a statement on the validation of imaging biomarkers, which states that the coefficient of variation (CV) of a biomarker should be 15% or less [16]. The CV is defined as the standard deviation of a metric relative to its means, with the notion that the observed variability in the metric should reflects measurement errors, so that means that the CV * 1.96 is equal to CoR. Thus, the CV for our results can be computed as CoR/1.96. Given our CoR results, we need to conclude that the performance of the method is not yet good enough for use in the clinical setting. Still, we think that the method has value for research, as the limited precision can be compensated by increasing the sample size in research trials that use this metric.

This research showed test–retest reliability for hemodynamic parameters in perforating arteries in the BG at 3 T, which can be used to calculate the expected effect size. Currently, no literature on the expected effect size in case of e.g. SVD on 3 T MRI is available for such power calculation. The best approximation we can make is based on meta-analysis data of vPI measurements obtained in a larger intracranial artery using transcranial Doppler, i.e. the middle cerebral artery in healthy controls vs. patients with vascular dementia [17]. The average difference in vPI from that meta-analysis study was 46%. Alternatively, Schnerr et al. found a difference in the (flow) pulsatility index of the perforating arteries in the basal ganglia of approximately 53% with ageing on 7 T [8]. Detecting an increase of 50% in vPI of the perforating arteries in the BG with a power of 0.8 and a significance level of 0.05, would require a sample size of only 18 subjects (9 controls vs. 9 patients), given the vPI (0.43) and standard deviation of 0.16 (maximum from Table 3) measured in this study. This is a very reasonable number, suggesting that studying the effects of SVD on the perforating arteries with 3 T MRI is feasible.

From the Bland–Altman plots with repositioning, we see that, although not significant, both *V*_mean_ and vPI have lower mean values with later scans. This is consistent with the expectation that subjects might relax more over time, which is supported by the (not significant either) lower heart rate for the two later scans.

### Limitations

This study had some limitations. First, planning of the 2D-PC slice was done manually, and we did not do a test–retest measurement of the planning. The manual planning was performed by only one experienced operator (JZ) for all included subjects. In clinical practice, however, multiple different operators plan subjects, which could increase the differences in outcome parameters between repeated scans with repositioning. A way to limit the impact of the operator on the planning is to plan MRI neuro scans automatically [18].

Second, relatively large numbers are needed for a robust estimation of the limits of agreement of repeated measurements as follows: for 90% confidence of estimating the standard deviation within 20% of its true values, one needs 35 subjects [10]. Ideally we would have preferred to increase the confidence to 95% within of 10% deviation from the true value, but this would require approx. 200 subjects, which was not feasible.

The third limitation concerned the relative low temporal resolution of the 2D-PC measurements. A lower temporal resolution leads to flattening of the velocity curve and thus to underestimation of the vPI. However, the temporal resolution is also limited in ultra-high field [2]. 3 T MRI vPI values were not systematically lower than the 7 T MRI vPI values for the matched analysis as shown in a previous study [9].

To conclude, this study shows that cerebral perforating artery velocity and pulsatility measurements can be performed at 3 T MRI with reasonable reliability. This confirms that functional vascular parameters in small perforating vessels that are relevant for clinical research in, e.g. SVD can be measured on 3 T. The obtained results can be used to perform power calculations for designing such clinical studies at 3 T.

## Abbreviations

BG: Basal ganglia
CoR: Coefficient of repeatability
CV: Coefficient of variation
DC: Dice coefficient
LoA: Limits of agreement
MRI: Magnetic resonance imaging
*N*_detected_: Number of detected vessels
ROI: Region of interest
SNR: Signal to noise ratio
SPSS: Statistical package of social sciences
SVD: Small vessel disease
vPI: Velocity pulsatility index
*V*_mean_: Mean velocity
2D-PC: Two-dimensional phase contrast
1.5/3/7 T: 1.5/3/7 Tesla field strength MRI
